# Recent Shift in Age Pattern of Dengue Hemorrhagic Fever, Brazil

**DOI:** 10.3201/eid1410.071164

**Published:** 2008-10

**Authors:** Maria Gloria Teixeira, Maria Conceição N. Costa, Giovanini Coelho, Mauricio L. Barreto

**Affiliations:** Federal University of Bahia, Salvador, Bahia, Brazil (M.G. Teixeira, M.C.N. Costa, M.L. Barreto); Ministry of Health, Brasilia, Brazil (G. Coelho)

**Keywords:** dengue fever, dengue hemorrhagic fever, age shift, Brazil, children, letter

**To the Editor:** Brazil is responsible for >60% of reported cases of dengue fever (DF) in the American region (a designation of the Pan American Health Organization, which includes all of North, Central and South America) (*1*). The epidemiologic characteristics of dengue diseases in Brazil differ from those described in Southeast Asia. In Brazil, the incidence of DF and dengue hemorrhagic fever (DHF) is highest in adults. By contrast, in Southeast Asia, DHF cases predominate and occur more often in children than in adults (*2*,*3*).

We describe a preliminary report of a shift in age group predominance that was observed during the 2007 countrywide dengue epidemic in Brazil. The Hospital Information System is the source of data describing the distribution of DHF cases from January 1998 through December 2007 (*4*). In Brazil almost all patients with a diagnosis of DHF are hospitalized. This country has promulgated the use of the World Health Organization’s DHF case definitions (International Classification of Diseases, 10th revision – A91). In the 2007 epidemic, a larger than normal proportion of cases were DHF (2,706), more than twice the largest number of such cases reported in previous years. Moreover, in 2007 >53% of cases were in children <15 years of age; during 1998–2006, the predominance of DHF cases were in the 20- to 40-year age group (Appendix Figure). During 1998–2006, the percentage of DHF cases in children varied from 9.5% (in 1998) to 22.6% (in 2001).

Of the 2,706 DHF cases in 2007, 1,710 (63.2%) were reported from the northeast region; 1,119 (65.4%) of these were in children <15 years of age. The southeast region had the next largest number (558), accounting for 20.6% of all reported cases; however, only 26.2% were in children. Other regions with cases, central-west and northern, reported no substantial change in age distribution compared with earlier years. Among the 9 states in northeastern Brazil, DHF predominance in children was observed in Maranhao (609 cases; 92.0% in children), Rio Grande do Norte (97 cases and 77.6%), Pernambuco (316 cases and 67.0%), and Ceara (197 cases and 48.0%).

The change in age distribution of cases in 2007 is unique in the modern history of dengue in Brazil and requires an explanation. Dengue 1 and 2 viruses, which were introduced in the 1990s, generated epidemics of DF characterized by a low incidence of DHF, predominantly in adults. With the introduction of dengue 3 virus in 2000–2001, DF epidemics of greater magnitude were observed, with a slightly larger fraction of DHF cases. Differences in the epidemiologic patterns in Southeast Asia and the American region have been attributed to genetic resistance in black populations and to underreporting of DHF cases, among other factors (*2*). These factors seem insufficient to explain the sudden change observed; should it persist—as it has in Venezuela, Colombia, Central America, and Cuba—this change may bring dengue in Brazil to a pattern closer to that of Southeast Asia (*2*). This change in epidemiologic pattern of dengue cases supports calls for improvement in design of dengue surveillance studies to include, where possible, population-based serologic studies. These epidemiologic changes also serve as an alert to health authorities in the American region to update their healthcare services to provide agile, opportune, and good quality care for patients, particularly children, with DHF, to reduce deaths.

## Supplementary Material

Appendix Figure Number of hospitalizations for dengue hemorrhagic fever by age group and year of occurrence, Brazil, 1998-2007.
