# Supplementary figures and images for: Recent Shift in Age Pattern of Dengue Hemorrhagic Fever, Brazil
Source: Emerg Infect Dis. 2008 Oct;14(10):1663. doi: 10.3201/eid1410.071164 (PMC2609867; doi:10.3201/eid1410.071164)

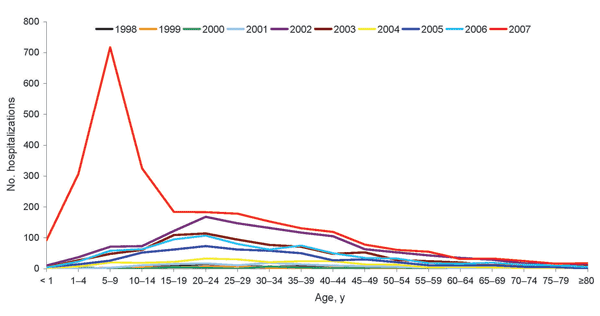

Supplement: Appendix Figure — Number of hospitalizations for dengue hemorrhagic fever by age group and year of occurrence, Brazil, 1998-2007. [file 07-1164_app-s1.gif]
